# The Community Health Assessment Program in the Philippines (CHAP-P) diabetes health promotion program for low- to middle-income countries: study protocol for a cluster randomized controlled trial

**DOI:** 10.1186/s12889-019-6974-z

**Published:** 2019-06-03

**Authors:** Gina Agarwal, Ricardo N. Angeles, Lisa Dolovich, Janusz Kaczorowski, Jessica Gaber, Dale Guenter, Floro Dave Arnuco, Hilton Y. Lam, Lehana Thabane, Daria O’Reilly, Rodelin M. Agbulos, Rosemarie S. Arciaga, Jerome Barrera, Elgie Gregorio, Servando Halili, Norvie Jalani, Fortunato Cristobal

**Affiliations:** 1 Department of Family Medicine, McMaster University, 1280 Main St W, Hamilton, Ontario L8S 4L8 Canada; 2 Leslie Dan Faculty of Pharmacy, University of Toronto, 144 College Street, Toronto, Ontario M5S 3M2 Canada; 3 Department of Family and Emergency Medicine, University of Montreal and CRCHUM, 850 Saint-Denis St, Montreal, Quebec, H2X 0A9 Canada; 4 School of Medicine, Ateneo de Zamboanga University, La Purisima St, 7000 Zamboanga City, Philippines; 5 Institute of Health Policy and Development Studies, University of the Philippines Manila, One Adriatico Place Tower One, Adriatico Street Corner Pedro Gill Street, 1000 Manila, Philippines; 6 Department of Health Research Methods, Evidence, and Impact, McMaster University, 1280 Main St W, Hamilton, Ontario L8S 4L8 Canada; 7 Zamboanga City Health Office, Pettit Barracks, 7000 Zamboanga City, Philippines; 8 Zamboanga Medical Research Foundation, Zamboanga City, Philippines; 9 Graduate School, Ateneo de Zamboanga University, La Purisima St, 7000 Zamboanga City, Philippines; 10 Department of Health, Zamboanga Peninsula, 7000 Zamboanga City, Philippines

**Keywords:** Cluster randomized trial, Low- to middle-income countries (LMICs), Philippines, Diabetes mellitus, Hypertension, Health promotion, Disease prevention

## Abstract

**Background:**

Type 2 diabetes is increasing globally, with the highest burden in low- to middle-income countries (LMICs) such as the Philippines. Developing effective interventions could improve detection, prevention, and treatment of diabetes. The Cardiovascular Health Awareness Program (CHAP), an evidence-based Canadian intervention, may be an appropriate model for LMICs due to its low cost, ease of implementation, and focus on health promotion and disease prevention. The primary aim of this study is to adapt the CHAP model to a Philippine context as the Community Health Assessment Program in the Philippines (CHAP-P) and evaluate the effect of CHAP-P on glycated hemoglobin (HbA1c) compared to a random sample of community residents in control communities.

**Methods:**

Six-month, 26-community (13 intervention, 13 control) parallel cluster randomized controlled trial in Zamboanga Peninsula, an Administrative Region in the southern Philippines. Criteria for community selection include: adequate political stability, connection with local champions, travel feasibility, and refrigerated space for materials. The community-based intervention, CHAP-P sessions, are volunteer-led group sessions with chronic condition assessment, blood pressure monitoring, and health education. Three participant groups will be involved: 1) Random sample of community participants aged 40 or older, 100 per community (1300 control, 1300 intervention participants total); 2) Community members aged 40 years or older who attended at least one CHAP-P session; 3) Community health workers and staff facilitating sessions. Primary outcome: mean difference in HbA1c at 6 months in intervention group individuals compared to control. Secondary outcomes: modifiable risk factors, health utilization and access (individual); diabetes detection and management (cluster). Evaluation also includes community process evaluation and cost-effectiveness analysis.

**Discussion:**

CHAP has been shown to be effective in a Canadian setting. Individual components of CHAP-P have been piloted locally and shown to be acceptable and feasible. This study will improve understanding of how best to adapt this model to an LMIC setting, in order to maximize prevention, detection, and management of diabetes. Results may inform policy and practice in the Philippines and have the potential to be applied to other LMICs.

**Trial registration:**

ClinicalTrials.gov (NCT03481335), registered March 29, 2018.

**Electronic supplementary material:**

The online version of this article (10.1186/s12889-019-6974-z) contains supplementary material, which is available to authorized users.

## Background

As of 2013, an estimated 382 million people are living with type 2 diabetes globally, with the large majority of these individuals living in low- to middle-income countries (LMICs) [1]. It is estimated that 45.8% of individuals with diabetes are undiagnosed, with 83.8% of those undiagnosed being from LMICs [2]. The number of people with diabetes is expected to increase significantly by 2035, with the projected increase higher in low (108%) and low-middle income (60%) countries compared to upper-middle (51%) and high income (28%) countries [1]. For LMICs, one of the challenges of managing the impact of diabetes is developing effective and low-cost interventions to prevent or delay the onset of type 2 diabetes that can be successfully implemented, scaled up, and sustained [3]. Several studies have demonstrated the cost-effectiveness of early diagnosis and management of diabetes through the use of opportunistic screening and risk assessment screening tools [4, 5]. Therefore, adopting and testing an effective low-cost intervention that enhances early diagnosis and management of diabetes shows promise for LMICs.

In 2013, the Philippines, considered an LMIC, had an overall diabetes prevalence of 6.0%, an estimated 1.7 million people with undiagnosed diabetes, and 54,535 diabetes-related deaths [6]. The Philippines is estimated to have over 6 million people with diabetes by the year 2035 [1]. The Philippines has begun implementation of the World Health Organization Package of Essential Noncommunicable Disease Interventions for primary care settings in low resource areas [7], created the National Centre for Disease Prevention and Control in 2000 including a diabetes-specific office, and has listed reduction of mortality and morbidity from lifestyle-related diseases such as diabetes as one of the goals in the “National Objectives for Health 2005-2010” [8]. However, there are currently some gaps in the areas of detection and treatment for diabetes. For example, though screening kits and medications are available at no cost, the case detection for new cases of diabetes is poor, the diabetes registry is poorly maintained, and the medications often get left unused in the stockrooms of local health centres (Dr M.A. Mabolo, Philippine Department of Health, personal communication, June 16, 2014). Knowledge about diabetes is also a gap in the Philippines; in a study in one region of the Philippines the mean score for diabetes knowledge among people diagnosed with diabetes was only 43% [9].

The Cardiovascular Health Awareness Program (CHAP) intervention model may be particularly suited to LMICs due to its low cost, implementability, and focus on population-based health promotion and disease prevention. CHAP is a community-based, primary care-centred, volunteer-led, free of charge, cardiovascular disease risk assessment and blood pressure monitoring program, which is combined with health education sessions for community-dwelling older adults [10]. A large community cluster randomized controlled trial in Canada demonstrated that the CHAP intervention resulted in a statistically significant 9% reduction in annual hospital admissions due to stroke, heart failure, and heart attacks in people aged 65 and over at the population [11]. The CHAP has been successfully expanded to include a diabetes risk assessment component in the Community Health Awareness of Diabetes (CHAD) program and other community adaptations [12, 13].

The Community Health Assessment Program for the Philippines (CHAP-P) was based on a formal partnership between universities in the Philippines (Ateneo de Zamboanga University School of Medicine) and Canada (McMaster University Department of Family Medicine) with guidance from a Project Advisory Committee composed of collaborators and researchers from Canada, the Philippines, Peru, Thailand, Tunisia, and the UK. The CHAP-P intervention was developed through a multi-stage study that combined specific elements of the CHAP and CHAD, adapting the intervention to be more appropriate for LMICs in general and the local setting of communities in Southwestern Philippines (Zamboanga Peninsula) in particular. Zamboanga Peninsula was chosen as the program site for this initiative because it exemplifies underprivileged regions in many LMICs in terms of geographical isolation, poverty, and scarce health resources where low-cost, community-owned health programs – such as CHAP-P – are urgently needed.

This multi-stage research project is culminating in a parallel cluster randomized controlled trial (RCT), the protocol of which is the focus of this paper. The primary aim of this RCT is to determine the effects of the CHAP-P intervention on HbA1c levels among a random sample of community residents 40 years of age and older, compared to a random sample of community residents in control communities under usual care. The secondary aims are to determine CHAP-P’s effectiveness compared to usual care in impacting: i) modifiable lifestyle risk factors for developing type 2 diabetes; ii) self-reported health utilization and access to care; iii) diabetes detection and management indicators in clusters (screening rates, initiation of medical management, hospital admissions, and mortality due to diabetes and its complications); and iv) program cost-effectiveness and cost-utility.

### Theoretical framework and development approach

**Fig. 1:**
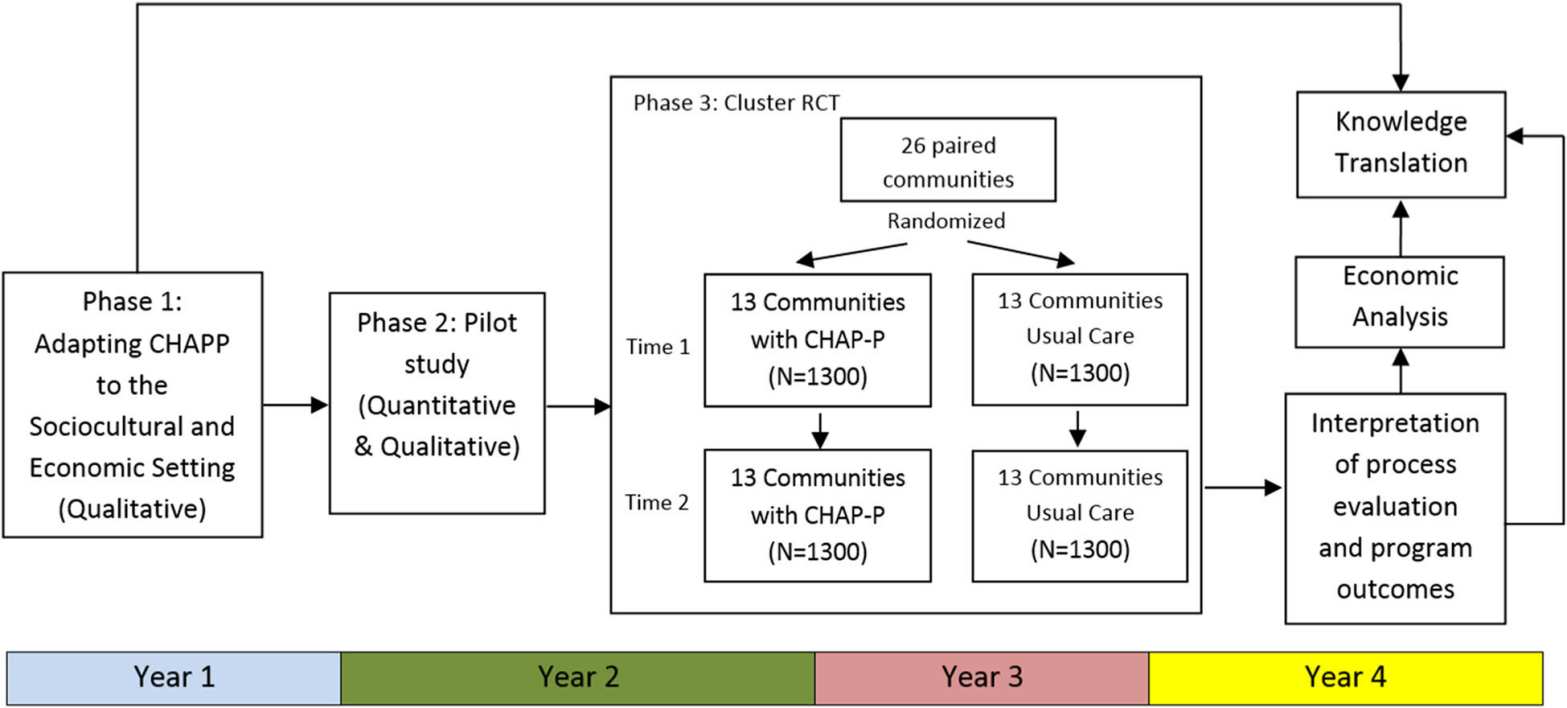
Overview of the CHAP-P Research Program

The overall project is a three-phase mixed methods evaluation. Phase 1 was a qualitative community scan that examined the sociocultural, economic, and health service context in the Zamboanga Peninsula, Philippines in order to adapt the CHAP intervention for best fit. Phase 2 was a three-stage pilot study to finalize assessment tools and evaluation methods, and identify potential problems with implementation in preparation for the RCT. Finally, Phase 3 is the RCT described in this paper. See Fig. 1 for an overview of the overall project design.

Greenhalgh and colleagues’ model of diffusion of innovation [14] was used to guide the adaptation of CHAP to the Philippines context during Phase 1. Our project is also guided by the Integrated Innovation approach, which posits that one can create a synergistic effect when addressing an issue or challenge by combining social, business, and scientific innovations [15]. Social innovations can bring scientific solutions to a local setting, while business innovations can deliver them at an affordable price point. The Integrated Innovation approach will be combined with the knowledge-to-action process, which depicts the relationship between knowledge creation and action steps to promote the application of knowledge (in our case the generation of CHAP-P) and evaluation of the success of the actions taken [16].

## Methods/design

### Setting

Zamboanga Peninsula is an Administrative Region of the Philippines on the island of Mindanao in the southern Philippines consisting of three provinces (Zamboanga del Norte, Zamboanga del Sur, and Zamboanga Sibugay) and two independent cities (Isabela and Zamboanga City). In 2012, this region had the fourth highest poverty incidence of the 17 regions of the Philippines (33.7%) [17]. Communities are separated by sea and mountains, with several ethnic and linguistic groups spread throughout the region and connected by inconsistent transportation and communication systems. The provinces and cities are broken down into municipalities, and the municipalities are broken into the smallest administrative districts in the Philippines, known as *barangays*, which are small villages or neighbourhoods. Each municipality is classified with an income class ranging from first (the highest income class) to sixth (the lowest) based on their average income in a four-year period. For the evaluation purposes, we will be recruiting 100 randomly selected residents aged 40 and over from each of 26 barangays (communities).

### Design

**Fig. 2:**
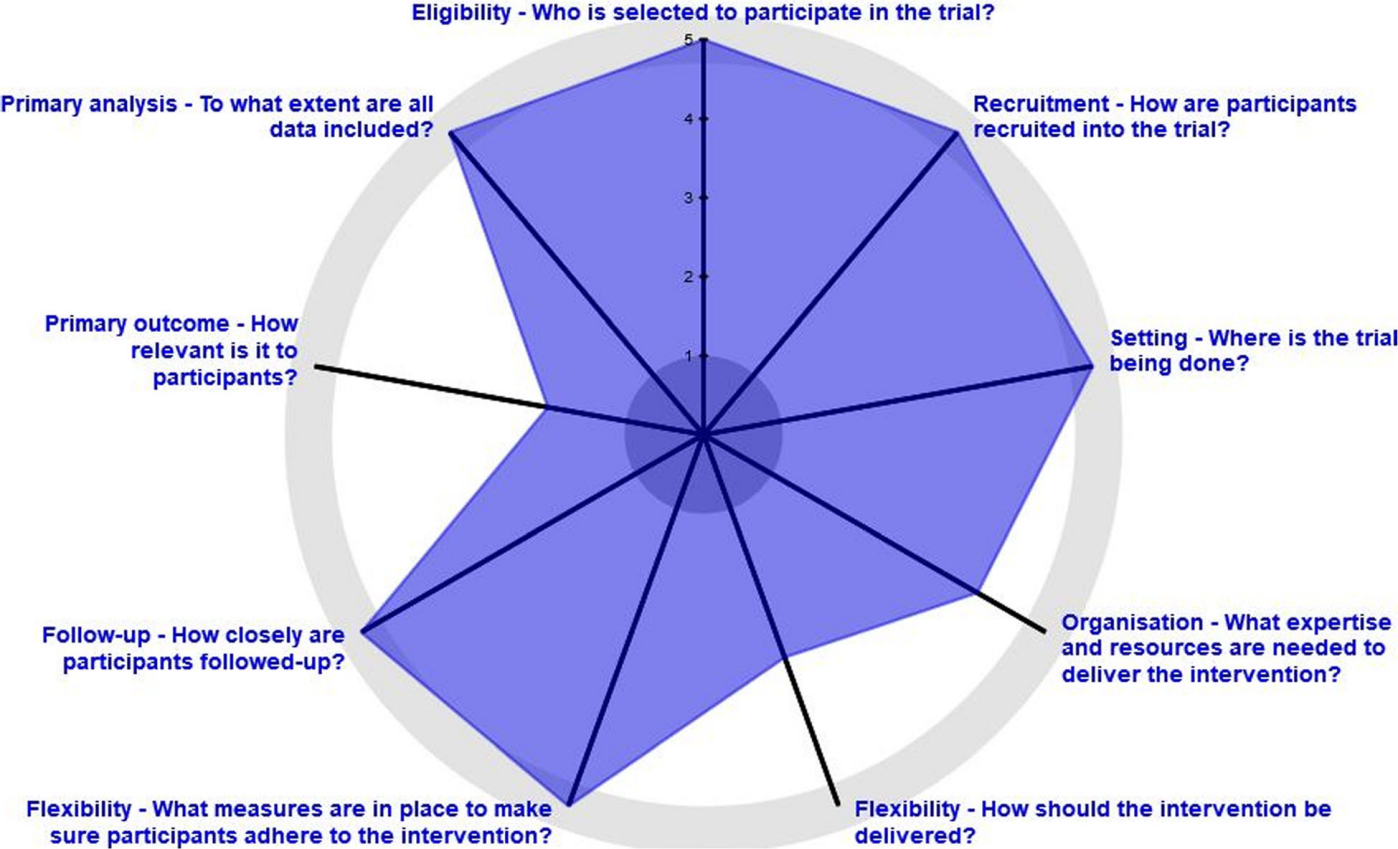
PRECIS Diagram

This study is a 26-community parallel open-label cluster randomized controlled trial. A community cluster design was chosen as CHAP-P is intended as a community-level intervention. Potential communities will be stratified by province, population size, income class, and type (urban versus rural). Thirteen barangays will receive the CHAP-P sessions and will be considered intervention communities, while the other 13 communities will receive care as usual and will be considered control communities. This will be done through paired randomization with staggered starts. Communities will be selected and randomized by the local program manager. Criteria for community selection include: security, connection with a local champion, feasibility for travel, and facility with refrigerated space for the HbA1c kits. Barangays will be paired based on municipality, population size, and similarity in the setting (i.e., religion, being under the same health district providing health services, and distance from the municipal centre). This will be done based on the advice of the local health workers and leaders in the municipalities and cities. Allocation will be done by computer generated randomization. Measurements of individual participants will be taken as a repeated cross-sectional sample at baseline and at 6 months. The Pragmatic-Explanatory Continuum Indicator Summary 2 (PRECIS-2) [18] was used to make design decisions based on the pragmatism of the trial (see Fig. 2). Reporting will follow the CONSORT 2010 statement: extension for cluster randomized trials [19]; reporting of this protocol follows the SPIRIT 2013 checklist [20], see Additional file 5.

### Participant recruitment

There will be three participant groups in the study. First, 100 participants will be randomly selected and recruited from each of the 26 barangays involved in the trial for a community survey. Households will be chosen via door-to-door systematic random sampling conducted by research staff. Within selected households, individuals 40 years of age or older will be eligible to participate in the survey. If there is more than one eligible and willing individual within a household, the last-birthday selection method [21] will be used to choose a single individual to participate. If there are no eligible or willing participants within a household, the team will move on to the next household based on the systematic random sampling procedure. A member of the research staff team will ask individuals selected to participate in the study to provide consent, and the survey will be conducted with consenting individuals (See Additional files 1, 2, 3 and 4 for all consent forms). Participants will then be asked to go to a community location for the HbA1c test and to be provided with a small token of appreciation. The survey participants are not necessarily the same individuals who will attend the CHAP-P sessions in intervention communities, though entire communities will be invited to the CHAP-P sessions.

The second participant group are those that attend the CHAP-P sessions. CHAP-P participants are community residents aged 40 years of age or older. As part of participating in the CHAP-P sessions, individuals give consent to participate in the study and for the information collected during the sessions to be linked with municipal/city health office records.

The final participant group is the Barangay Health Workers (BHWs) and other Lead Local Organization (LLO) staff that will be involved in facilitating the CHAP-P sessions. They will be recruited through convenience sampling with research team members inviting those who have been involved with the CHAP-P intervention. Those BHWs and other LLO staff that consent to be part of the evaluation will be invited to participate in focus groups or key informant interviews, depending on their role and availability.

Due to the nature of the intervention as a community-wide health promotion program, this is an open label trial. However, community survey participants are not necessarily aware of the ongoing trial, community assignments, and study group allocation of their community.

### Outcomes

#### Primary outcome and measure

The primary outcome is the mean difference in HbA1c at 6 months in a random sample of individuals from the random sample of individuals from the intervention barangays compared to the control barangays. HbA1c will be tested at a community location at baseline and 6 months, after the participants have completed the community survey in their homes.

#### Secondary outcomes and measures

There are a number of secondary outcomes which will evaluate the mean differences between intervention and control groups at 6 months. These outcomes, whether they pertain to cluster or individual participant level, and their measures and sources are listed in Table 1.

**Table 1:** Secondary Outcomes

Outcome	Level	Measure, Source
Physical activity	Individual	International Physical Activity Questionnaire (IPAQ) [22] and modified CP@clinic survey [13]
Medication compliance	Individual	Survey questions (self-reported)
Blood pressure	Individual	Physical measurements
Risk factors for diabetes	Individual	Finnish Diabetes Risk Score (FINDRISC) [23] on community survey
Risk behaviours for diabetes / cardiovascular issues (activity, diet, smoking, alcohol, stress)	Individual	Elements of the Health Awareness and Behaviour Tool (HABiT) on community survey (self-reported)
Knowledge about diabetes and hypertension	Individual	Elements of the HABiT on community survey (self-reported)
Perceived concern and understanding of risk	Individual	Elements of the HABiT on community survey (self-reported)
Confidence in behaviour change	Individual	Elements of the HABiT on community survey (self-reported)
Number of community residents newly diagnosed with diabetes	Cluster	Rural Health Unit databases
^a^Hospital admission rates due to diabetes and diabetes-related conditions, hypertension, myocardial infarction (MI), stroke, congestive heart failure	Cluster	Central Department of Health database
^a^Mortality rates due to diabetes and diabetes-related conditions, hypertension, MI, stroke, congestive heart failure based on ICD-10 codes	Cluster	Regional Field Health Surveillance Information Systems

^a^Hospital admission rates and mortality rates will be measured for both the 12 months before and the 12 months after the CHAP-P implementation, and will be divided by the mid-year population estimates

#### Community process evaluation and fidelity checks

A community process evaluation will also be undertaken during the project in order to monitor the implementation of the CHAP-P intervention to assess for any problems or process issues during or after the implementation of the project. Monthly reports from communities, monthly observational fidelity checklists from research assistants, and qualitative focus groups/interviews will be analyzed for this component of the evaluation.

#### Cost-effectiveness and cost-utility

A cost-effectiveness analysis will be conducted comparing the program cost of implementing CHAP-P and healthcare resource utilization costs to percentage reduction in HbA1c. A cost-utility analysis will also be conducted to determine the cost of the program and healthcare resource utilization costs per quality-adjusted life year (QALY) gained, using the EuroQol-5 dimension-5 level (EQ-5D-5 L) [24, 25] as the indicator of quality of life.

### Data collection and management

Community survey participants will be interviewed in their homes by trained research staff at baseline and 6 months. Questionnaires will be completed on paper and later entered into a REDCap [26] database by trained research staff. This survey was adapted from the measure used in the CP@clinic program in Canada [13] and includes questions from other validated questionnaires as well as physical measurements such as blood pressure, height, weight, and waist circumference. The questions include: demographics; knowledge about diabetes and cardiovascular health; risk factors and behaviours, including the Finnish Diabetes Risk Calculator (FINDRISC) [23]; quality of life using the EQ-5D-5 L [24, 25]; perceived confidence; perceived concern and understanding of risk; self-efficacy to improve health behaviours; physical activity using the International Physical Activity Questionnaire (IPAQ) [22]; and health utilization and access. The WatchBP Office Target will be used to measure blood pressure as it was found to show the most reliable results based on our pilot study [27]. This community survey will be completed at baseline and at 6 months and will use an open-cohort design.

After completing the community survey in their homes, these participants will be invited to a community location where they will have their glycated hemoglobin (HbA1c) tested using the A1CNow + point-of-care device, which is certified by the National Glycohemoglobin Standardization Program [28]. HbA1c testing will be conducted at baseline and 6 months, following the community survey at each time point.

Focus group discussions will be held at 6 months for study participants, BHWs, and other LLO staff using a standardized focus group guide consisting of open-ended questions primarily focused on identifying the barriers and facilitators to implementing CHAP-P. Further sources of data include monthly community reports from the communities, monthly observational checklists by research assistants, and record review of the CHAP-P session databases and Rural Health Unit databases.

For CHAP-P session participants, the FINDRISC, blood pressure, and other physical measurement data collected as part of the CHAP-P sessions will be included in the evaluation.

Paper data will be stored in a locked cabinet in a locked institutional office. Electronic data will be stored in an encrypted program (REDCap) or in password-protected files on a secure institutional network. Study data will be anonymized. For the community survey and CHAP-P session data, after the full data set is collected (including the HbA1c test results), data will be anonymized. For the qualitative data, once transcripts are produced from the interviews and focus groups, identifiers of participants will be removed.

### Intervention

**Fig. 3:**
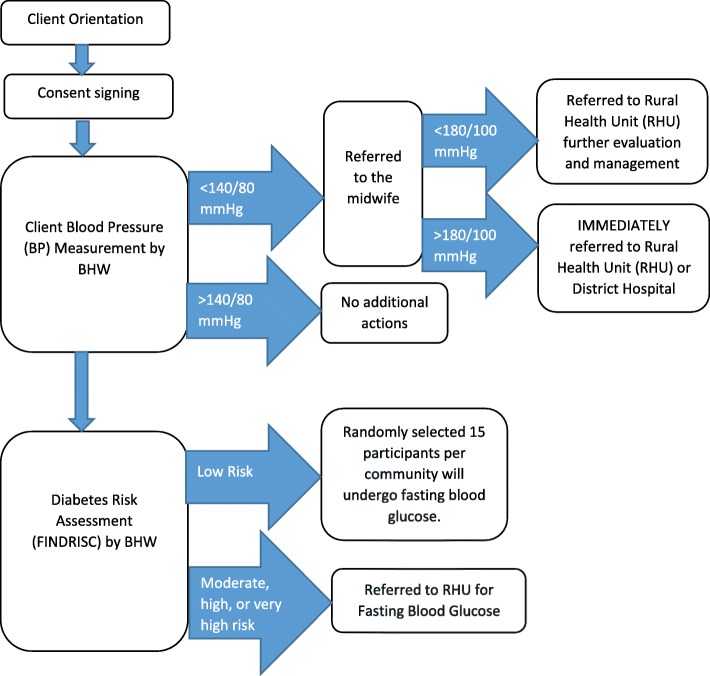
CHAP-P Intervention

The 13 intervention barangays will receive the CHAP-P sessions. The intervention will occur as follows (see Fig. 3). First, residents will be invited to attend the CHAP-P sessions, which are facilitated by Barangay Health Workers (BHWs), who are trained local volunteers. The BHW role is a voluntary position accredited by local health boards, which provides primary health care service to the barangays [29]. BHWs will receive program-specific in-person training at the start of CHAP-P, with refresher sessions during their work with the program. During the CHAP-P sessions, the BHWs will collect participants’ consent, measure blood pressure, collect other physical measurements (height, weight, waist circumference), and collect participant information to determine clients’ risk of diabetes using the FINDRISC [23]. All data will be collected through an electronic REDCap mobile app database [26] via a tablet computer, which was found to be the most accurate and acceptable choice of data collection method for BHWs during the pilot stages [30].

Based on findings during the assessment, BHWs will educate the CHAP-P participants on diabetes, cardiovascular risk factors, and healthy lifestyles, providing educational materials that have been adapted for a local context (including materials such as pamphlets, comic strips, and videos). Those CHAP-P participants whose FINDRISC scores indicate diabetes risk (moderate: score of 12–14, high: score of 15–20, or very high: score of > 20) will be referred to the Rural Health Unit for fasting blood glucose testing. Those who have blood pressure greater than 140/80 mmHg will be referred to the midwife, with those with blood pressure over 180/100 mmHg being immediately referred to the Rural Health Unit or District Hospital, and those with blood pressure under 180/100 mmHg being referred to the Rural Health Unit with less immediacy, for further evaluation and management. Those with other specific risk factors such as low physical activity, high salt intake, or smoking will be given further health education and referred to appropriate local programs. CHAP-P sessions will continue to be held twice a month in intervention communities, and residents will be encouraged to continue attending for ongoing follow-up and monitoring. The monthly observational fidelity checklists from research assistants will be undertaken to improve adherence to intervention protocols.

### Data analysis

#### Quantitative outcomes

The baseline characteristics will be analyzed using descriptive statistics reported by group as mean (standard deviation) or median (first quartile, third quartile) for continuous variables, and count (percentage) for categorical variables. The analysis of all outcomes to compare the groups will follow intention-to-treat principle. We will use multiple imputation to handle missing data. We will use Generalized Estimating Equations (GEE) to make comparisons between intervention and control communities, assuming an exchangeable correlation structure [31]. GEE will allow us to model the correlation of outcomes within communities. All results will be reported as estimates of effect, corresponding 95% confidence interval and associated *p*-values. All p-values will be reported to three decimal places with those less than 0.001 reported as *p* < 0.001. All analyses will be performed using SAS 9.4 (Cary, NC) or Stata 11 (College Station, TX). See Table 2 for the statistical analysis plan.

**Table 2:** Statistical Analysis Plan

Research Question	Outcomes and Measures	Participant Group(s)	Time Point	Hypotheses	Method of Analysis
1. What is the difference in HbA1C levels between communities implementing CHAP-P and those not implementing CHAP-P?	• Mean difference in HbA1c (A1C Now+ point-of-care device)	Community survey participants	T_0_, T_6_	Mean HbA1c level will have a greater decrease in intervention communities over control communities	Independent samples t-test (intervention vs control), One-way ANOVA (subgroup analysis), Generalized estimating equations (adjust for clustering effect)
2. What is the difference in modifiable lifestyle risk factors between communities implementing CHAP-P and those not implementing CHAP-P?	• Physical activity (IPAQ) • Medication compliance (self-report, survey question) • Blood pressure (BP; WatchBP Office Target device) • Risk factors for diabetes (FINDRISC) • Risk Behaviours for Diabetes / Cardiovascular Issues (activity, diet, smoking, alcohol, stress; self-report, measured with elements of the Health Awareness and Behaviour Tool [HABiT]) • Knowledge about Diabetes and Hypertension (self-report; measured with elements of the HABiT) • Perceived Concern and Understanding of Risk (self-report; measured with elements of the HABiT) • Confidence in Behaviour Change (self-report; measured with elements of the HABiT)	Community survey participants (all outcomes) CHAP-P session participants (change in BP over time; diabetes risk score)	*Survey* T_0_, T_6_ *Session* BP: each session Diabetes risk score: T_0_, T_6_	In intervention versus control communities, physical activity, medication compliance, knowledge, perceived concern and understanding of risk, and confidence in behaviour change will have more of an increase, blood pressure, diabetes risk score, and risk behaviours will have more of a decrease In CHAP-P session participants, blood pressure will decrease with more length of time involved in the program; diabetes risk score will decrease pre to post.	Independent samples t-test (intervention vs control), One-way ANOVA (subgroup analysis), Generalized estimating equations (adjust for clustering effect), Hierarchical linear modelling (BP over time)
3. What is the difference in diabetes-specific outcomes between communities implementing CHAP-P and those not implementing CHAP-P?: (a) screening rates for diabetes, (i.e., rates of newly diagnosed cases of type 2 diabetes), (b) initiating medication treatment for diabetes management, and (c) hospital admission rates and mortality due to diabetes (and its complications), hypertension, myocardial infarction, stroke, and congestive heart failure? (d) self-reported health utilization and access	• a. N community residents newly diagnosed with diabetes (Rural Health Unit databases) • b. N community residents newly initiating medication treatment • c. Hospital admission rates due to diabetes and diabetes-related conditions, hypertension, MI, stroke, CHF (central Department of Health database) • d. Mortality rates due to diabetes and diabetes-related conditions, hypertension, MI, stroke, CHF based on ICD-10 codes (Regional Field Health Surveillance Information Systems) • e. Availability of health care services in community and whether there is a place they go when sick or need health advice (2 items; self-report)	a., b., c., d. N/A – administrative records representing intervention and control communities e. Community survey participants	a., b. T_6_ (for 6 months of program) c., d. T_−12_, T_12_ (for 12 months before & after program, divided by mid-year population estimates) e. T_0_, T_6_	Intervention over control communities will have more residents newly diagnosed with diabetes, more residents newly initiating medication treatment for diabetes, a lower hospital admission rate due to diabetes and related conditions, and a lower mortality rate due to diabetes and related conditions	Generalized estimating equations (linear and Poisson Model, adjust for clustering effect)
4. What is the cost-effectiveness and cost-utility of CHAP-P compared to usual care?	• Program cost (actual) • Healthcare resource utilization costs (self-report, survey) • Percentage reduction in HbA1c (A1C Now+ point-of-care device) • Quality of life (EQ-5D-5 L) • Quality-Adjusted Life Years (QALYs) (calculated from EQ-5D-5 L)	Community survey participants (healthcare resource utilization, HbA1c, quality-of-life)	T_0_, T_6_	Not appropriate for this type of outcome	*Cost-effectiveness:* percentage decrease in HbA1c *Cost-utility:* cost per QALY (based on local EQ-5D-5 L)
(Implementation Outcomes)	• Community process evaluation – interviews and focus groups, monthly community reports, observational fidelity checklists	Interview and focus group participants (BHWs, CHAP-P session participants, LLO staff)	T_6_	Not appropriate for this type of outcome	Thematic analysis

#### Qualitative analysis

Transcripts will be cleaned and data will be summarized using thematic analysis (open coding axial coding, selective coding). QSR International NVivo 11 qualitative analysis software [32], will be used to store and manage qualitative data.

#### Economic analysis

Percentage decrease in participant HbA1c will be the measure of effectiveness used in the cost-effectiveness analysis. Cost per QALY will be calculated as the cost-utility measure; QALYs will be computed based on local EQ-5D-5 L values. Both cost-effectiveness and cost-utility analyses will include overall program cost measures and participant health resource utilization and cost thereof.

#### Power and sample size

CHAP-P is a community-wide intervention and community sizes vary from 3000 to 20,000 residents. We are using paired randomization, therefore intervention and control community pairs need to have relatively similar sizes. Our sample size for individuals was calculated based on a mean difference of HBA1c of 0.2% (SD = 1.09) with standard parameters (alpha = 0.05, power = 0.80). This required a sample size of 520 per arm. Based on our pilot, the intraclass correlation coefficient (ICC) was 0.006. We increased our ICC to 0.01 which inflated our sample size to 1034 per arm. We have opted to take 26 (13 pairs of intervention: control) communities with 100 residents per community giving a total sample size of 2600 residents (1300 per arm).

## Discussion

This study will provide a robust evaluation of a community diabetes program in the Philippines. Though the country is committed to preventing and treating lifestyle-related diseases such as diabetes, the level of commitment to implement programs and the amount of diabetes-related activities already being implemented varies greatly among communities [8]. Some community-based programs for type 2 diabetes tested in other LMICs including elements such as educational sessions, lifestyle instruction, and self-monitoring have shown significant positive outcomes including lowering weight, waist circumference, fasting plasma glucose levels, and HbA1c [33, 34]. Diabetes self-management programs and other community-based interventions are being implemented across the Philippines, yet large-scale experimental studies are still a gap in the literature [9, 35–37]. This study will help fill that gap.

A major strength of this study is the multi-phase, multi-year nature of the overall research program. The intervention was based on the evidence-based CHAP model from Canada, though needed to be adapted for the local context. Phase 1, the qualitative community scan, and Phase 2, piloting the elements, were vital to integrate the components and building a diabetes intervention that would make sense in the context of the Zamboanga Peninsula, Philippines. The mixed methods design included throughout the research program is another strength, with qualitative data included in this RCT to help explain and interpret quantitative results. Initially, a stepped-wedge cluster RCT was planned rather than a parallel cluster RCT, yet instead of solving issues of logistical constraints [38], it introduced some - the stepped-wedge design would have been more expensive and more time-consuming. Due to the variation in communities across the Philippines, the focus on a community-level approach is a key element in making a program such as CHAP-P work. The integration of perspectives from multiple key stakeholders throughout the process solidifies communities’ buy-in to the project. Finally, the health economics component of this study will provide policymakers and funders the information they need to decide whether implementation of a program such as CHAP-P is cost-effective for their communities.

Though we combined many elements to fit the program to the setting, there are still some limitations that warrant consideration. First, recruitment for CHAP-P sessions can be difficult, particularly within urban areas, and as a community intervention it is important to reach a substantial portion of the community. To improve penetration within the communities, we have decided to ensure the CHAP-P sessions rotate between *puroks* (a subdivision of a barangay) rather than having them in the same location week-by-week. Second, the mobile HbA1c test kit can be difficult to work with, as it only lasts for approximately 5 minutes in the heat and humidity of the Philippines. Due to that constraint, all HbA1c tests are now completed at a common community location; however, those locations still need refrigeration for the test kits, so access to refrigerated space is an inclusion criterion for communities. Finally, during the pilot studies there were some security concerns in the region that limited travel of the research team, so something similar may occur during the RCT implementation. This may affect the ability of the research team to visit communities for monthly fidelity checks, yet the intervention will be able to continue as BHWs can continue facilitating CHAP-P sessions in their local communities.

This study has the potential to improve diabetes detection, management, and prevention in the Philippines and similar LMICs. The results from this study will be shared with policymakers (municipal, provincial, regional, national) in the Philippines and our research partners in other LMICs and the Global Alliance for Chronic Diseases. Results will also be published in relevant conference and journals to disseminate our findings to other researchers and policy makers planning to implement an out-of-the-box program for diabetes detection, management, and prevention.

## Additional files

Additional file 1: Consent Form – Community Survey Participant. (DOCX 165 kb)

Additional file 2: Consent Form – CHAP-P Session Participant. (DOCX 169 kb)

Additional file 3: Consent Form – Community Resident Focus Group. (DOCX 164 kb)

Additional file 4: Consent Form – BHW. (DOCX 173 kb)

Additional file 5: SPIRIT 2013 Checklist. (DOC 123 kb)

## Abbreviations

ADZU: Ateneo de Zamboanga University, Philippines
BHW: Barangay Health Worker
CHAD: Community Health Awareness of Diabetes
CHAP: Cardiovascular Health Awareness Program
CHAP-P: Community Health Assessment Program in the Philippines
CIHR: Canadian Institutes of Health Research
EQ-5D-5 L: EuroQol-5 dimension-5 level (quality of life measure)
FINDRISC: Finnish Diabetes Risk Score (measure)
GACD: Global Alliance of Chronic Diseases
HABiT: Health Awareness and Behaviour Tool
HbA1c/A1C: Glycated hemoglobin
HiREB: Hamilton Integrated Research Ethics Board, Canada
IDRC: International Development Research Centre
IPAQ: International Physical Activity Questionnaire (measure)
LMIC: Low- to middle-income country
QALY: Quality-adjusted life year
RCT: Randomized controlled trial
