# Supplementary material for: The Community Health Assessment Program in the Philippines (CHAP-P) diabetes health promotion program for low- to middle-income countries: study protocol for a cluster randomized controlled trial
Source: BMC Public Health. 2019 Jun 3;19:682. doi: 10.1186/s12889-019-6974-z (PMC6547510; doi:10.1186/s12889-019-6974-z)
Supplement: Supplementary file 1 — Consent Form – Community Survey Participant. (DOCX 165 kb) [file 12889_2019_6974_MOESM1_ESM.docx]

**CONSENT FOR COMMUNITY SURVEY AND HBA1C**

**Project Title:** Community Health Assessment Program Philippines (CHAP-P)

**Principal Investigators:** Fortunato Cristobal, MD, MHPEd, MPH (Ateneo de Zamboanga University, Philippines), Nominated Principal Investigator, Philippines (63) 062 310-4316

Gina Agarwal, MBBS, PhD (McMaster University, Canada), Lisa Dolovich, BScPhm, PharmD MSc (McMaster University, Canada), Janusz Kaczorowski, PhD (University of Montreal, Canada), Ricardo Angeles, MD, MPH, MHPEd, PhD (McMaster University, Canada/Ateneo de Zamboanga University, Philippines)

**Sponsor:** Canadian Institutes of Health Research (CIHR)

**Participant Information SheeT**

**COMMUNITY SURVEY and Hba1c**

Prior/After to implementing the Community Health Assessment Program Philippines (CHAP-P) in your community, you are being invited to participate in a survey to assess the knowledge and practices of residents 40 years and older in your community regarding risk of diabetes and heart diseases, as well as assess your risk factors you have related to your lifestyle. This is a research study, and you are not under any obligation to take part. This project seeks to understand whether CHAP-P will be/was able to help community residents like yourselves in achieving better knowledge in diabetes and heart disease and improve your health practices.

**WHY IS THIS STUDY BEING DONE?**

The main goal of CHAP-P is to help improve the awareness of community residents 40 years and older regarding their risk of diabetes and heart disease. To check if we have been successful in achieving this goal, we would like to assess the knowledge and health practices of residents like yourselves before and after implementing CHAP-P in your community.

WHAT IS THE PURPOSE OF THIS STUDY?

The purpose of this study is to assess if CHAP-P will be helpful in rural settings in communities like the Philippines. We would therefore want to know if CHAP-P significantly improved the knowledge, awareness and health practices of residents 40 years of age and older regarding diabetes and heart health.

**WHAT WILL MY RESPONSIBILITIES BE IF I TAKE PART IN THE STUDY?**

If you volunteer to participate in this study, you will be asked to participate in this survey that will take no more than 30 minutes in length. This interview will be scheduled at a time convenient for you to discuss what you know about diabetes and heart disease, and your practices that help improve or worsen your risk of having diabetes and heart health. We will also check blood sugar control level (HBA1c).

**HOW MANY PARTICIPANTS WILL BE IN THIS STUDY?**

In this study we expect to have over 100 people taking part in interviews in 26 communities (barangays) in Tampilisan, Zamboanga del Norte (Region IX),Philippines.

**WHAT ARE THE POSSIBLE RISKS AND DISCOMFORTS?**

To assess the blood sugar level, you will feel some pain in the finger prick for blood extraction. You may refuse this procedure. It is possible that during the interview, some participants may feel uncomfortable sharing their information. You can stop participating at anytime if you feel uncomfortable.

**WHAT ARE THE POSSIBLE BENEFITS FOR ME AND/OR FOR SOCIETY?**

There are no direct benefits to participants. Participants will also know whether their blood sugar is normal. Based on the results of this study, we will know if the CHAP-P was helpful to residents in your community and support the development of CHAP-P to improve its implementation in your community as well as other areas in Philippines.

**IF I DO NOT WANT TO TAKE PART IN THE STUDY, ARE THERE OTHER CHOICES?**

It is important for you to know that you can choose not to take part in the study. You are free to withdraw from this study at any time without any consequences.

**WHAT INFORMATION WILL BE KEPT PRIVATE?**

Your data will not be shared with anyone except with your consent or as required by law. All personal information such as your name, address, and phone number will be removed from the data and will be replaced with a number. A list linking the number with your name will be kept in a secure place, separate from your data, and will be destroyed within 10 years of publishing this study. The electronic data, with identifying information removed will be securely stored in an encrypted file, on a password protected computer in a limited access area. Information will only be accessible by the research team. You may ask to review your information.

The information collected will be kept secure and private. When we examine the data, we will look at large amounts of information. No single person’s records or personal information will ever be reported. The research study has received ethics approval at ADZU-Research Ethics Board at *tel.nos.991-0871*.

**CAN PARTICIPATION IN THE STUDY END EARLY?**

You have the option of removing your data from the study. You may also refuse to answer any questions you don’t want to answer and still remain in the study. At any time, you can discontinue your participation by not participating in interviews, or withdraw your consent to have your information shared by calling the CHAP-P Project Coordinator **Dr. Floro Dave Arnuco** at *tel.nos.310-4316*.

**WILL I BE PAID TO PARTICIPATE IN THIS STUDY?**

If you agree to take part, you will not receive a token of appreciation for the time taken to participate in this study.

**WILL THERE BE ANY COSTS?**

Your participation in this study will not involve any additional costs to you.

**Consent to Participate**

To indicate consent to the following options, please initial in the box:

| (place initials here) | |
| --- | --- |
|  | **I agree to participate in a CHAP-P survey during (______).** You can withdraw from the study at any time.  **I consent for my blood sugar and HBA1c level to be checked during (______).** You can withdraw from the study at any time. |

I understand that whether I do or do not decide to participate in any aspect of the program or research will not affect the care that I receive. I understand that my participation is voluntary and I am not required to provide any information or answer any questions that I do not wish to answer. I understand that my information will be kept secure and private and will not be used for any purpose to which I have not explicitly consented. I understand that, at any time, I can discontinue my participation by no participating in interviews, or withdraw my consent to have my information shared by calling the CHAP-P Project Coordinator **Dr. Floro Dave Arnuco** at *tel.nos.310-4316*.

All the information I needed to make an informed decision was given to me and all of my questions were answered.

I understand the information on this consent form and I will receive a signed copy.

**I agree to participate and have placed my initials to indicate agreement with the options above.**

**Name (*please print*) Name of Person Obtaining Consent (*please print*)**

**Signature Date Signature Date**

**If you have any questions about the study** now or later, please contact **Dr. Floro Dave Arnuco,** Project Coordinator at *tel.nos.310-4316*.

**If you have any questions regarding your rights** as a study participant, you may contact **ADZU-Research Ethics Board** at *tel.nos.991-0871*.
