# Supplementary material for: The Community Health Assessment Program in the Philippines (CHAP-P) diabetes health promotion program for low- to middle-income countries: study protocol for a cluster randomized controlled trial
Source: BMC Public Health. 2019 Jun 3;19:682. doi: 10.1186/s12889-019-6974-z (PMC6547510; doi:10.1186/s12889-019-6974-z)
Supplement: Supplementary file 2 — Consent Form – CHAP-P Session Participant. (DOCX 169 kb) [file 12889_2019_6974_MOESM2_ESM.docx]

**CONSENT FOR COMMUNITY PARTICIPANT (CHAP-P SESSION)**

**Project Title:** Community Health Assessment Program Philippines (CHAP-P)

**Principal Investigators:** Fortunato Cristobal, MD, MHPEd, MPH (Ateneo de Zamboanga University, Philippines), Nominated Principal Investigator, Philippines (63) 062 310-4316

Gina Agarwal, MBBS, PhD (McMaster University, Canada), Lisa Dolovich, BScPhm, PharmD MSc (McMaster University, Canada), Janusz Kaczorowski, PhD (University of Montreal, Canada), Ricardo Angeles, MD, MPH, MHPEd, PhD (McMaster University, Canada/Ateneo de Zamboanga University, Philippines)

**Sponsor:** Canadian Institutes of Health Research (CIHR)

**Participant Information Sheet**

**COMMUNITY PARTICiPANT – CHAP-P SESSION**

Prior to implementing the Community Health Assessment Program Philippines (CHAP-P) in your community, you are being invited to participate in the CHAP-P sessions being done in your community. This is a research study, and you are not under any obligation to take part. This project seeks to understand whether CHAP-P will be/was able to help community residents like yourselves in achieving better knowledge in diabetes and heart disease and improve your health practices.

**WHY IS THIS STUDY BEING DONE?**

The main goal of CHAP-P is to help improve the awareness of community residents 40 years and older regarding their risk of diabetes and heart disease and improve their health practices.

WHAT IS THE PURPOSE OF THIS STUDY?

The purpose of this study is to assess if CHAP-P will be helpful in rural settings in communities like the Philippines. We would therefore want to know if CHAP-P: 1) can be adapted to rural communities in the Philippines; 2) will significantly improve the knowledge, awareness and health practices of residents 40 years of age and older regarding heart health and diabetes.

**WHAT WILL MY RESPONSIBILITIES BE IF I TAKE PART IN THE STUDY?**

If you volunteer to participate in this study, you will be asked to participate in this CHAP-P session. These sessions will assess your risk of developing diabetes and heart disease. In addition, we will also check your blood pressure, weight, height, and waist circumference. If you have a high blood pressure or a high risk of developing diabetes, we will refer you to the Rural Health Unit for further check-up and blood sugar testing.

**HOW MANY PARTICIPANTS WILL BE IN THIS STUDY?**

In this study we expect to have as many participants in the CHAPP sessions in each of the 13 communities across the Zamboanga Peninsula (Region IX), Philippines.

**WHAT ARE THE POSSIBLE RISKS AND DISCOMFORTS?**

The blood pressure check may be uncomfortable since your blood pressure will be taken 3 times by our blood pressure machine. If you require blood sugar testing, you will feel some pain in the finger prick for blood extraction. You may refuse this procedure. It is possible that during the interview, some participants may feel uncomfortable sharing their information. You can stop participating at anytime if you feel uncomfortable.

**WHAT ARE THE POSSIBLE BENEFITS FOR ME AND/OR FOR SOCIETY?**

Participants may benefit from learning the important things to know and practice to have a healthy heart and avoid having diabetes. Participants will also know whether their blood sugar and blood pressure is normal and take further action in case it is detected to be elevated.Further this information will support the development of CHAP-P to improve its implementation in your community as well as other areas in Philippines.

**IF I DO NOT WANT TO TAKE PART IN THE STUDY, ARE THERE OTHER CHOICES?**

It is important for you to know that you can choose not to take part in the study. You are free to withdraw from this study at any time without any consequences.

**WHAT INFORMATION WILL BE KEPT PRIVATE?**

Your data will be shared with the Municipal/City Health Office with your consent. This is done so that the staff of the Municipal/City Health Office can monitor your health, and if needed, follow-up with you in case they have health education/promotion programs that can help you. The research study has received ethics approval at **ADZU-Research Ethics Board** at *tel.nos.991-0871*.

**CAN PARTICIPATION IN THE STUDY END EARLY?**

You have the option of removing your data from the study. You may also refuse to answer any questions you don’t want to answer and still remain in the study. At any time, you can discontinue your participation by not participating in interviews, or withdraw your consent to have your information shared by calling the CHAP-P Project Coordinator **Dr. Floro Dave Arnuco** at *tel.nos.310-4316*.

**WILL I BE PAID TO PARTICIPATE IN THIS STUDY?**

There will be no payment for participants in this study.

**WILL THERE BE ANY COSTS?**

Your participation in this study will not involve any additional costs to you.

**Consent to Participate**

To indicate consent to the following options, please initial in the box:

| (place initials here) | |
| --- | --- |
|  | **I agree to participate in a CHAP-P sessions during (______).** You can withdraw from the study at any time.  **I consent for my blood pressure to be taken during (______).** You can withdraw from the study at any time.  **I consent for my blood sugar to be taken during (______). You can withdraw from the study at any time.**  **I consent for my information to be shared with the Municipal/City Health Office (______).** You can withdraw from the study at any time. |

I understand that whether I do or do not decide to participate in any aspect of the program or research will not affect the care that I receive. I understand that my participation is voluntary and I am not required to provide any information or answer any questions that I do not wish to answer. I understand that my information will be kept secure and private and will not be used for any purpose to which I have not explicitly consented. I understand that, at any time, I can discontinue my participation by no participating in interviews, or withdraw my consent to have my information shared by calling the CHAP-P Project Coordinator **Dr. Floro Dave Arnuco** at *tel.nos.310-4316*.

All the information I needed to make an informed decision was given to me and all of my questions were answered.

I understand the information on this consent form and I will receive a signed copy.

**I agree to participate and have placed my initials to indicate agreement with the options above.**

**Name (*please print*) Name of Person Obtaining Consent (*please print*)**

**Signature Date Signature Date**

**If you have any questions about the study** now or later, please contact **Dr. Floro Dave Arnuco,** Project Coordinator at *tel.nos.310-4316*.

**If you have any questions regarding your rights** as a study participant, you may contact **ADZU-Research Ethics Board** at *tel.nos.991-0871*.
